# Expression of alternatively spliced HTLV-1 mRNAs is influenced by mitosis and by a novel cis-acting regulatory sequence

**DOI:** 10.1186/1742-4690-12-S1-P90

**Published:** 2015-08-28

**Authors:** Ilaria Cavallari, Francesca Rende, Marion K Bona, Joanna Sztuba-Solinska, Stuart FJ LeGrice, Genoveffa Franchini, Donna M D'Agostino, Vincenzo Ciminale

**Affiliations:** 1 Department of Surgery, Oncology and Gastroenterology, University of Padova, Italy; 2 Leidos Biomedical Research, Inc., Frederick National Laboratory, Maryland, USA; 3 Basic Research Laboratory, National Cancer Institute, Frederick, Maryland, USA; 4 Animal Models and Retroviral Vaccines Section, National Cancer Institute, Bethesda, Maryland, USA; 5 Department of Biomedical Sciences, University of Padova, Italy; 6 Istituto Oncologico Veneto-IRCCS, Padova, Italy

## 

Human T-cell leukemia virus type 1 (HTLV-1) expression depends on the concerted action of Tax, which enhances transcription of the viral genome, and Rex, which favours expression of incompletely spliced mRNAs. In the present study we investigated the influence of Rex on the nucleo-cytoplasmic partitioning of the complete set of alternatively spliced HTLV-1 mRNAs. Analyses of cells transfected with Rex-wild type and Rex-knock out HTLV-1 molecular clones using splice site-specific qRT-PCR revealed that mRNAs encoding the p30Tof, p13, and p12/8 proteins were Rex-dependent, while the p21rex mRNA was Rex-independent. These findings provide a rational explanation for the intermediate-late temporal pattern of expression of the p30tof, p13, and p12/8 mRNAs described in previous studies. Cell cycle block experiments indicated that mitosis partially bypasses the requirement for Rex to express Rex-dependent HTLV-1 transcripts. All the Rex-dependent mRNAs contained a 75-nucleotide intronic region that was able to increase nuclear retention and degradation in the absence of other viral sequences. Selective 2’-hydroxyl acylation (SHAPE) analysis revealed that this sequence formed a stable hairpin structure. The findings reported in this study add a layer of complexity to the mechanisms controlling the expression of alternatively spliced HTLV-1 mRNAs and suggest a link between the cycling properties of the host cell and the pattern of viral expression.

